# The relationship between animal flesh foods consumption and rheumatoid arthritis: a case-control study

**DOI:** 10.1186/s12937-022-00800-1

**Published:** 2022-07-30

**Authors:** Elahe Hatami, Mobina Aghajani, Makan Pourmasoumi, Farahnaz Haeri, Behnoosh Boozari, Saeed Nezamoleslami, Cain C. T. Clark, Shokufeh Nezamoleslami, Reza Ghiasvand

**Affiliations:** 1 Department of Exercise Physiology, Sport Medicine Research Centre, Sport Sciences Research Institute, Tehran, Iran; 2 Neuromusculoskeletal Research Center, Iran University of Medical Sciences, Tehran, Iran; 3 Gastrointestinal and Liver Diseases Research Centre, Guilan University of Medical Sciences, Rasht, Iran; 4 Food Security Research Center, Isfahan University of Medical Sciences, Isfahan, Iran; 5 Department of Community Nutrition, School of Nutrition and Food Science, Isfahan University of Medical Sciences, Isfahan, Iran; 6 Department of Clinical Nutrition, School of Nutrition and Food Science, Isfahan University of Medical Sciences, Isfahan, Iran; 7 Department of Nutrition, Science and Research Branch, Islamic Azad University, Tehran, Iran; 8 Centre for Intelligent Healthcare, Coventry University, Coventry, CV1 5FB UK

**Keywords:** Diet, Rheumatoid arthritis, Animal flesh foods, Incidence, Case-control

## Abstract

**Background:**

Rheumatoid arthritis (RA) is a chronic, systemic inflammatory, and debilitating autoimmune illness. The objective of the present study was to evaluate the relationship between animal flesh foods consumption and rheumatoid arthritis.

**Methods:**

Meat consumption was assessed by using a semi-quantitative Food Frequency Questionnaire (168 items) in a case-control study of 297 subjects (100 newly diagnosed cases and 197 healthy controls). An expert rheumatologist diagnosed patients based on the American College of Rheumatology definitions, 2010. Multivariate logistic regression, adjusted for lifestyle and nutritional confounders, was used to evaluate the relationship between dairy consumption and rheumatoid arthritis.

**Results:**

Participants with greater consumption of fish and seafood were less likely to have RA (OR 0.52; 95% CI 0.27–0.98). Conversely, a higher processed meat intake was associated with increased odds of RA (OR 3.45; 95% CI 1.78–6.68). However, no significant association was found between red meats and poultry consumption and the risk of RA in the fully adjusted model.

**Conclusions:**

The present study suggests an inverse association between fish and seafood consumption and the risk of RA. On the contrary, a higher amount of processed meat intake was associated with increased odds of RA. However, further studies are warranted to confirm the veracity of our findings.

## Background

Rheumatoid arthritis (RA) is a chronic, systemic inflammatory, and debilitating autoimmune illness that is more common in women and affects about 1% of the world’s population [1, 2]. The main characteristics of RA include chronic inflammation of the synovial tissue, progressive destruction of cartilage, bone erosion, pain, and eventually, permanent joint damage [3, 4]. The disease can have extensive negative effects on quality of life and life expectancy, increases healthcare use, contribute to a loss of work, and represents a substantial economic burden, both individually and societally [5, 6]. Although the cause of RA is not yet fully understood, according to various reports, genetic and environmental factors are implicated in the expression of the disease [7]. Environmental risk factors that can play a role in the progression and/or development of RA include smoking, socioeconomic, stress, viruses, and diet [8, 9]. Diet represents a modifiable risk factor that can act as a disease trigger or an inflammatory response moderator. Studies on diet and risk of RA may help to identify modifiable and preventative factors [10–12]. Several studies have shown correlations between dietary components and changes in inflammation and disease activity in patients with rheumatoid arthritis [13]; indeed, diets containing animal products (e.g., dairy and red meat) may exacerbate RA due to their pro-inflammatory properties [14]. The prevalence of autoimmune diseases is higher in Western countries, which can, at least partly, be attributed to, or influenced by, dietary habits [15]. In fact, the Western diet, which is typically high in saturated and trans fats, refined carbohydrates, sugar-sweetened drinks, and a low ratio of omega-3: omega-6 fatty acids, increases inflammation and the risk of RA. The main feature of the Western diet is the high consumption of animal proteins and red meat, which, in a nested case-control study, was reported to increase the risk of inflammatory polyarthritis [16, 17]. However, another case-control study in China, which was conducted on 968 patients with RA diagnosed according to American College of Rheumatology criteria and 1037 matched healthy controls, found no association between red meat intake and the risk of RA [18]. Although a number of studies have shown a reduced risk of RA with increased fish consumption [19], there are numerous contradictory findings that require further study [18, 20]. Indeed, due to the controversies between studies and the lack of evidence in Iran, the present study was conducted to investigate the relationship between animal flesh foods consumption and RA.

## Materials and methods

### Study population

Details on the methodology of the present study have been previously reported [21]. Briefly, in this case-control study, newly diagnosed RA patients (maximum 12 months since diagnosis) who attended a rheumatology clinic in Isfahan, Iran, were recruited. The presence of RA was confirmed by a rheumatologist based on the American College of Rheumatology 2010 classification criteria [22]. Eligibility criteria included: lack of a history of chronic diseases, such as cardiovascular diseases, renal disease, liver diseases, or cancer; not pregnant or lactating; not consuming alcohol; have no known food allergies or adhering to special lifestyle/diets. Furthermore, we excluded participants who did not complete the Food Frequency Questionnaire (FFQ), did not answer more than 35 items of the questionnaire, or whose energy intake showed extremely high or low (energy intake 800–4200 kcal/day) [23]. All participants gave written informed consent. This study was approved by the Research Council and the Ethics Committee of Isfahan University of Medical Sciences (IR. MUI.Research.REC.1398.120).

### Dietary assessment

Diet was assessed using data from a semi-quantitative Food Frequency Questionnaire (FFQ) containing the frequency and the amount of dietary intake. The FFQ was validated previously in Iranian populations [24]. The questionnaire included 168 food items, and information was collected through interviews by a trained dietitian. The frequency of meat consumption (red meat, poultry, fresh fish, canned fish, processed meats, and organ meats) was assessed with a questionnaire response regarding the average consumption of various foods over the past year, ranging from never to three times or more per day.

### Assessment of other variables

Anthropometric data were assessed through standard methods. Body weight was measured by a digital scale, to an accuracy of 100 g, with participants unshod and in light clothes. Height was measured using a non-stretchable measuring tape, with participants in a standing position and unshod. Body mass index (BMI) was calculated through the following formula: weight in Kg divided by height in m squared. Also, waist circumference was obtained from the midpoint region between the undersides of the rib and the upper part of the pelvis using a non-stretch plastic measuring tape.

Data regarding demographic features and socioeconomic status (SES) of the participants were collected through a self-reported questionnaire. Variables included: age, sex, education (below high school diploma; diploma; diploma to bachelor’s degree; on top of bachelor’s degree), type of occupation, home status (owner/tenant), home type (apartment/villa), family size (≤4/> 4), family the disease history as well as smoking status.

Physical activity rate was assessed by the use of a validated and reliable short form of the International Physical Activity Questionnaire (Short IPAQ) [25]. Participants responded to seven questions regarding low, intermediate, and high levels of physical activity. Then physical activity rate of each subject was measured by metabolic equivalent task (MET) - minutes/day.

### Statistical analyses

All analyses were conducted using SPSS (version 21.0, SPSS Inc., Chicago, Illinois, USA). The normal distribution of data was examined by the Kolmogorov-Smirnov test. To explore the difference in general characteristics, macronutrients, and animal meat intake between case and controls, independent t-test or Chi-square were applied based on the nature of the data. Multi-variable adjusted models were used to investigate the association between different types of animal meat consumption and risk of RA in three different models; crude model with no adjustment; model 1, which was controlled for age, sex, BMI, and physical activity; and model 2 with additional adjustment for total energy intake, smoking, socioeconomic status (SES), drug use and family the disease history. A *P*-Value of less than 0.05 was considered, a priori, to represent statistical significance.

## Result

At the recruitment of study participants, 100 cases and 197 sex-matched controls were eligible based on the inclusion/exclusion criteria. Table 1 presents the general characteristics of study participants for each group. The mean age of cases and controls were 49.26 ± 12.6 and 40.88 ± 9.72y, respectively (*P*-*value*: < 0.001). There was no significant difference in the proportion of men/women between the case and control groups. The remaining variables, including BMI, family history of the disease, drug use percentage, smoking levels, total energy, protein and fat intake, and physical activity levels, were significantly higher in the case group than control, whilst a higher socioeconomic status, education, and carbohydrate intake were seen in the control compared to the case group.

**Table 1:** General characteristics of participants in case and control group

Variables	Case	Control	***P***-value
**Age(year)**	49.26 ± 12.6	40.88 ± 9.72	< 0.001
**Sex, female (%)**	81%	76.6%	0.37
**BMI (kg/m2)**	26.2 ± 4.35	24.82 ± 3.2	0.006
**Socioeconomic status (SES) b**	11.94 ± 3.47	13.57 ± 3.45	< 0.001
**Smoker**	17%	2%	< 0.001^a^
**Family disease history**	59%	4.09%	< 0.001^a^
**Drug use**	42%	1%	< 0.001^a^
**Education**			
**Lower than diploma**	50%	14.7%	< 0.001^a^
**Diploma**	33%	37%	
**Bachelor**	15%	31.5%	
**Higher than bachelor**	2%	16.8%	
**Energy intake (Kcal)**	2243.02 ± 598.55	2168 ± 687.3	0.365
**Carbohydrate (gr)**	258.29 ± 82	317.1 ± 116.63	0.015
**Protein (gr)**	62.09 ± 18.49	72.59 ± 28.24	< 0.001
**Fat (gr)**	98.57 ± 28	73 ± 32.32	< 0.001
**Physical activity (MET-min/week)**	4512.29 ± 6160	2372.74 ± 2055	< 0.001

Quantitative variables are shown as mean ± SD, and Qualitative variables as frequency (percentage)

*P*-values are calculated by independent *t*-test

*SD* Standard deviation, *CI* Confidence interval, *BMI* Body mass index

^a^For categorical data, *P*-values are assessed by Chi-square test

^b^Socioeconomic status (SES) score was evaluated based on education level of both subjects and the family head, job of both subjects and the family head, family size, home status and home type by using self-reported questionnaire

The mean and SD of different types of meat consumption, in both cases and controls, are shown in Table 2. There was a significant difference between cases and controls in terms of consuming processed meat, fish, and seafood; however, the amount of red meat, organ meat, and poultry intake was approximately the same in the case and control groups.

**Table 2:** Amount of meat intake in cases and controls

Variables	Case (***n*** = 100)		Control (***n*** = 197)		*P*-value^*^
	Mean	SD	Mean	SD	
**Red Meats**	23.20	38.25	16.74	17.56	0.09
**Proceed meats**	5.09	6.69	1.20	3.34	< 0.001
**Organ meats**	2.82	6.48	2.30	3.71	0.46
**Fish and sea foods**	2.30	3.71	8.69	7.99	< 0.001
**Poultry**	17.96	11.85	22.16	29.65	0.17

^*^P-value obtained from independent t-test

**Fig. 1:**
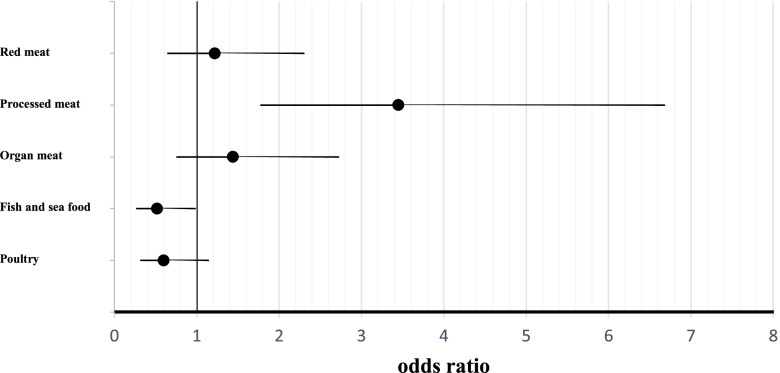
Full-adjustment model for the association between flesh meat group intake and odds of RA

Multi-variable adjusted OR for the risk of RA across the median of meat intake are shown in Table 3 and Fig. 1. Compared to those in the lower median, those in the upper median of processed meats consumption had a greater risk of RA in the crude model (OR 3.82; 95% CI 2.28–6.42). After potential confounders, including age, sex, BMI, waist circumference, and physical activity, were taken into account, the association remained significant (OR 3.83; 95% CI 2.18–6.72). In addition, after further adjustment for total energy intake, smoking, SES, drug use, and family the disease history, the association remained significant (OR 3.45; 95% CI 1.78–6.68). Conversely, participants in the upper median of fish and seafood intake had a 51% lower risk of RA compared to those in the lower median (OR 0.49; 95% CI 0.30–0.81). Such that, even after controlling for potential confounders, those with greater consumption of fish and seafood were less likely to have RA in both model 1 (OR 0.48; 95% CI 0.28–0.83) and model 2 (OR 0.52; 95% CI 0.27–0.98). Despite a marginally significant association between organ meats and odds of RA in both the crude (OR 1.62; 95% CI 0.99–2.63) and adjusted model 1 (OR 1.53; 95% CI 0.90–2.60), the relationship became non-significant in the fully adjusted model 2 (OR 1.44; 95% CI 0.76–2.72). Finally, no significant association was found between red meats and poultry and the risk of RA.

**Table 3:** Multi-variable adjusted models for risk of RA across median of Meat intake

Variables	Models	<Median	>Median
**Red meats**	Crude model	1	1.29 (0.80–2.09)
	Model 1	1	1.20 (0.71–2.03)
	Model 2	1	1.22 (0.65–2.30)
**Processed meats**	Crude model	1	3.82 (2.28–6.42)
	Model 1	1	3.83 (2.18–6.72)
	Model 2	1	3.45 (1.78–6.68)
**Organ meats**	Crude model	1	1.62 (0.99–2.63)
	Model 1	1	1.53 (0.90–2.60)
	Model 2	1	1.44 (0.76–2.72)
**Fish and sea food**	Crude model	1	0.49 (0.30–0.81)
	Model 1	1	0.48 (0.28–0.83)
	Model 2	1	0.52 (0.27–0.98)
**Poultry**	Crude model	1	0.74 (0.45–1.19)
	Model 1	1	0.75 (0.44–1.27)
	Model 2	1	0.60 (0.32–1.14)

Model 1: adjusted for age, sex and BMI, physical activity

Model 2: adjusted for model 1 plus total energy intake, smoking, SES, drug use and family the disease history

## Discussion

The present case-control study assessed the relationship between animal flesh food consumption and the risk of RA. The results of this study indicate an inverse association between fish consumption and RA. In addition, we noted a significant direct association between processed meat intake and an increased risk of RA, whilst the other variables in meat groups, including intake of red meat, poultry, and organ meat, did not show any significant association with the disease.

RA is a debilitating disease that adversely affects patients’ abilities to perform normal daily activities and incurs high economic and societal costs due to the negative effects it has on various aspects of patients’ lives, employability, life expectancy, and mortality [5, 6, 26]. In the present study, we showed that participants with higher consumption of fish were less likely to have RA. Concordant with our results, the association between fish consumption and a reduced risk of RA has been shown in many empirical studies [27, 28]. Indeed, evidence suggests that consumption of individual foods, including fish, might reduce the risk of RA. In a case-control study conducted by Rusell et al. [29] on 1889 patients and 2146 controls, the results showed that consumption of oily fish reduced the risk of RA while intake of fish oil supplements did not show a beneficial result. In another study of 32,000 women in 1987 and 1997 from the Swedish Mammography prospective Cohort, Giuseppe et al. [30] found that intake of 1–3 servings of fish per week reduced the risk of RA. However, the results of a case-control study conducted in China failed to find a significant association between fish consumption and the risk of RA [18]. The reason for underlining this discrepancy is unclear. It might be due to differences in dietary habits and the usual amount of intake, which is specific for people from various regions. For instance, the amount of seafood intake reported in this study is much higher compared to our study. The association between fish intake and the risk of RA found in the present study might be attributed to the anti-inflammatory properties of fish oil [31, 32]; however, this is a complex interaction, and other fatty acids could also exhibit pro- or anti-inflammatory activity [33, 34].

Our study showed that greater consumption of processed meat was related to an increased risk of RA. In this line, the EPIC-Norfolk study showed that individuals who ate higher levels of meat products (for instance, sausage) were more likely to develop polyarthritis [17]. Several studies have shown that processed meat can increase inflammatory mediators, including CRP [35–37]. Processed meat refers to products that are usually made from red meat and cured, salted, or smoked to improve the taste, color, and shelf life. Processed meats, such as sausages, have a high amount of fat (Often 50% by weight of sausage or more), SFA, and cholesterol [38]. In general, processed foods maybe increase the intake of saturated fats, cholesterol, salt, nitrite, heme iron, and polycyclic aromatic hydrocarbons, depending on methods used for food preparation [39]. Thus, the result might be due to these various components, knowing that they play an important role in oxidative stress [40].

In the present study, we failed to find any significant association between red meat and poultry consumption and the risk of RA. Similar to our findings, the results of a cohort study in the United States showed no association between red meat and poultry and the incidence of RA [33]. Another prospective cohort (Nurses’ Health Study) conducted on 82,063 women, which followed participants for more than 20 years, could not find a significant association between red meat intake and RA incidence [41]. Although some empirical studies indicated that greater red meat consumption is correlated with an increased risk of RA [42, 43] and put the finger on Arachidonic acid as one of the components of red meat that can trigger the production of inflammatory agents [44], recent investigations revealed a different role for this fatty acid [45–47]. In addition, meat also contains iron, which is known as a risk factor for oxidative stress; however, studies have shown that the increased risk of RA with meat consumption is independent of iron intake, and no association was found between iron consumption and RA [17]. Perhaps the difference in study results can be attributed to methodology differences, age groups, study populations, diet, and other lifestyle factors. Indeed, such reports in the literature may help to explain why our study did not detect an association between red meat and RA. In addition, since we did not measure the blood concentrations of the various components in participants, we could not say with certainty whether the effect of micronutrients and their role in RA.

The major strengths of this study are the recruitment of newly diagnosed patients, which reduces the likelihood of dietary changes since diagnosis. We controlled the effects of several potential confounders in the statistical analyses, which aided in our ability to provide more accurate interpretations of the data. Notwithstanding these strengths, our study had a number of limitations that should be considered, including we recruited a relatively small sample size and evaluated dietary intake by using a self-reported 1-year FFQ, which may increase the possibility of error in measuring dietary intake as it mainly depends on the memory of participants. Furthermore, the participants of this study were only from Isfahan, Iran; thus, we cannot necessarily extend these results to all Iranians or indeed different countries or ethnicities. Moreover, the participants’ age, which has been suggested to be a risk factor for RA, was significantly different between the case and control groups; however, we controlled and minimized the effect of this confounder on the findings through the multivariable adjustment models. Finally, it should be mentioned that our results may have been influenced by some potential confounders that were not evaluated, such as genetic factors and stress levels. Thus, considering the strengths and limitations of our study, we strongly recommend that large-scale investigations into the association between dietary factors, particularly red meat and fish, be conducted, including diverse ethnic samples.

## Conclusion

The present study suggests that a higher fish and seafood intake might be associated with a decreased risk of RA, while a greater amount of processed meat consumption may be associated with an increased odd of the disease. It is well known that patients who suffer from RA encounter numerous restrictions and difficulties; our results provide better insight into the association between RA and different types of meat foods and further highlight the importance of dietary assessment in this patient group. Nevertheless, further studies with higher sample sizes and more diverse ethnicities and geographical locations are recommended to confirm the veracity of these findings**.**

## Data Availability

The datasets generated and analyzed during the current study are not publicly available due to the institution’s policy but will be available through contacts with the corresponding author on reasonable requests.

## Abbreviations

RA: Rheumatoid Arthritis
IPAQ: International Physical Activity Questionnaire
SES: Socioeconomic status
BMI: Body mass index
WC: Waist circumference
FFQ: Food Frequency Questionnaire
MET: Metabolic Equivalent Task
ANOVA: One-way Analysis of Variance
PUFAs: Polyunsaturated fatty acids
CVD: Cardiovascular Diseases

## References

[1] Gibofsky A (2012). Overview of epidemiology, pathophysiology, and diagnosis of rheumatoid arthritis. Am J Manag Care.

[2] Simard JF, Costenbader KH, Hernán MA, Liang MH, Mittleman MA, Karlson EW (2010). Early life factors and adult-onset rheumatoid arthritis. J Rheumatol.

[3] Alwarith J, Kahleova H, Rembert E, Yonas W, Dort S, Calcagno M (2019). Nutrition interventions in rheumatoid arthritis: the potential use of plant-based diets. A Review. Front Nutr.

[4] Rosillo MA, Alarcón-de-la-Lastra C, Sánchez-Hidalgo M (2016). An update on dietary phenolic compounds in the prevention and management of rheumatoid arthritis. Food Funct.

[5] Eriksson JK, Johansson K, Askling J, Neovius M (2015). Costs for hospital care, drugs and lost work days in incident and prevalent rheumatoid arthritis: how large, and how are they distributed?. Ann Rheum Dis.

[6] Birnbaum H, Pike C, Kaufman R, Maynchenko M, Kidolezi Y, Cifaldi M (2010). Societal cost of rheumatoid arthritis patients in the US. Curr Med Res Opin.

[7] Khanna S, Jaiswal KS, Gupta B (2017). Managing rheumatoid arthritis with dietary interventions. Front Nutr.

[8] Gómez-Puerta JA, Gedmintas L, Costenbader KH (2013). The association between silica exposure and development of ANCA-associated vasculitis: systematic review and meta-analysis. Autoimmun Rev.

[9] Cutolo M (2007). Sex and rheumatoid arthritis: mouse model versus human disease. Arthritis Rheum.

[10] Oliviero F, Spinella P, Fiocco U, Ramonda R, Sfriso P, Punzi L (2015). How the Mediterranean diet and some of its components modulate inflammatory pathways in arthritis. Swiss Med Wkly.

[11] Cutolo M, Nikiphorou E (2018). Don’t neglect nutrition in rheumatoid arthritis!. RMD Open.

[12] Manzel A, Muller DN, Hafler DA, Erdman SE, Linker RA, Kleinewietfeld M (2014). Role of “Western diet” in inflammatory autoimmune diseases. Curr Allergy Asthma Rep.

[13] Adam O, Beringer C, Kless T, Lemmen C, Adam A, Wiseman M (2003). Anti-inflammatory effects of a low arachidonic acid diet and fish oil in patients with rheumatoid arthritis. Rheumatol Int.

[14] Turner-McGrievy GM, Wirth MD, Shivappa N, Wingard EE, Fayad R, Wilcox S (2015). Randomization to plant-based dietary approaches leads to larger short-term improvements in dietary inflammatory index scores and macronutrient intake compared with diets that contain meat. Nutr Res.

[15] Rudan I, Sidhu S, Papana A, Meng SJ, Xin-Wei Y, Wang W (2015). Prevalence of rheumatoid arthritis in low–and middle–income countries: a systematic review and analysis. J Glob Health.

[16] Philippou E, Nikiphorou E (2018). Are we really what we eat? Nutrition and its role in the onset of rheumatoid arthritis. Autoimmun Rev.

[17] Pattison DJ, Symmons DP, Lunt M, Welch A, Luben R, Bingham SA (2004). Dietary risk factors for the development of inflammatory polyarthritis: evidence for a role of high level of red meat consumption. Arthritis Rheum.

[18] He J, Wang Y, Feng M, Zhang X, Jin Y-B, Li X (2016). Dietary intake and risk of rheumatoid arthritis—a cross section multicenter study. Clin Rheumatol.

[19] Tedeschi SK, Bathon JM, Giles JT, Lin T-C, Yoshida K, Solomon DH (2018). Relationship between fish consumption and disease activity in rheumatoid arthritis. Arthritis Care Res.

[20] Sparks JA, O’Reilly ÉJ, Barbhaiya M, Tedeschi SK, Malspeis S, Lu B (2019). Association of fish intake and smoking with risk of rheumatoid arthritis and age of onset: a prospective cohort study. BMC Musculoskelet Disord.

[21] Nezamoleslami S, Ghiasvand R, Feizi A, Salesi M, Pourmasoumi M (2020). The relationship between dietary patterns and rheumatoid arthritis: a case–control study. Nutr Metab.

[22] Aletaha D, Neogi T, Silman AJ, Funovits J, Felson DT, Bingham CO (2010). 2010 rheumatoid arthritis classification criteria: an American College of Rheumatology/European league against rheumatism collaborative initiative. Arthritis Rheum.

[23] Hu FB, Rimm E, Smith-Warner SA, Feskanich D, Stampfer MJ, Ascherio A (1999). Reproducibility and validity of dietary patterns assessed with a food-frequency questionnaire. Am J Clin Nutr.

[24] Asghari G, Rezazadeh A, Hosseini-Esfahani F, Mehrabi Y, Mirmiran P, Azizi F (2012). Reliability, comparative validity and stability of dietary patterns derived from an FFQ in the Tehran lipid and glucose study. Br J Nutr.

[25] Lee PH, Macfarlane DJ, Lam TH, Stewart SM (2011). Validity of the international physical activity questionnaire short form (IPAQ-SF): a systematic review. Int J Behav Nutr Phys Act.

[26] Jacobsson L, Lindroth Y, Marsal L, Juran E, Bergström U, Kobelt G (2007). Rheumatoid arthritis: what does it cost and what factors are driving those costs? Results of a survey in a community-derived population in Malmö, Sweden. Scand J Rheumatol.

[27] Linos A, Kaklamanis E, Kontomerkos A, Koumantaki Y, Gazi S, Vaiopoulos G (1991). The effect of olive oil and fish consumption on rheumatoid arthritis-a case control study. Scand J Rheumatol.

[28] Shapiro JA, Koepsell TD, Voigt LF, Dugowson CE, Kestin M, Nelson JL (1996). Diet and rheumatoid arthritis in women: a possible protective effect of fish consumption. Epidemiology..

[29] Rosell M, Wesley A-M, Rydin K, Klareskog L, Alfredsson L, Group ES (2009). Dietary fish and fish oil and the risk of rheumatoid arthritis. Epidemiology..

[30] Di Giuseppe D, Crippa A, Orsini N, Wolk A (2014). Fish consumption and risk of rheumatoid arthritis: a dose-response meta-analysis. Arthritis Res Ther.

[31] Klek S, Mankowska-Wierzbicka D, Scislo L, Walewska E, Pietka M, Szczepanek K (2020). High dose intravenous fish oil reduces inflammation—a retrospective tale from two centers. Nutrients..

[32] Zadeh-Ardabili PM, Rad SK (2019). Anti-pain and anti-inflammation like effects of Neptune krill oil and fish oil against carrageenan induced inflammation in mice models: current statues and pilot study. Biotechnol Rep.

[33] Tateishi N, Kakutani S, Kawashima H, Shibata H, Morita I (2014). Dietary supplementation of arachidonic acid increases arachidonic acid and lipoxin A_4_ contents in colon, but does not affect severity or prostaglandin E_2_ content in murine colitis model. Lipids Health Dis.

[34] Tateishi N, Kaneda Y, Kakutani S, Kawashima H, Shibata H, Morita I (2015). Dietary supplementation with arachidonic acid increases arachidonic acid content in paw, but does not affect arthritis severity or prostaglandin E2 content in rat adjuvant-induced arthritis model. Lipids Health Dis.

[35] Chai W, Morimoto Y, Cooney RV, Franke AA, Shvetsov YB, Le Marchand L (2017). Dietary red and processed meat intake and markers of adiposity and inflammation: the multiethnic cohort study. J Am Coll Nutr.

[36] Azadbakht L, Esmaillzadeh A (2009). Red meat intake is associated with metabolic syndrome and the plasma C-reactive protein concentration in women. J Nutr.

[37] Ley SH, Sun Q, Willett WC, Eliassen AH, Wu K, Pan A (2014). Associations between red meat intake and biomarkers of inflammation and glucose metabolism in women. Am J Clin Nutr.

[38] Mozaffarian D, Micha R, Wallace S (2010). Effects on coronary heart disease of increasing polyunsaturated fat in place of saturated fat: a systematic review and meta-analysis of randomized controlled trials. PLoS Med.

[39] Rohrmann S, Linseisen J (2016). Processed meat: the real villain?. Proc Nutr Soc.

[40] Kim Y, Keogh J, Clifton P (2015). A review of potential metabolic etiologies of the observed association between red meat consumption and development of type 2 diabetes mellitus. Metabolism..

[41] Benito-Garcia E, Feskanich D, Hu FB, Mandl LA, Karlson EW (2007). Protein, iron, and meat consumption and risk for rheumatoid arthritis: a prospective cohort study. Arthritis Res Ther.

[42] Jin J, Li J, Gan Y, Liu J, Zhao X, Chen J (2021). Red meat intake is associated with early onset of rheumatoid arthritis: a cross-sectional study. Sci Rep.

[43] Fraser GE (2003). Diet, life expectancy, and chronic disease: studies of Seventh-Day Adventists and other vegetarians.

[44] Stamp LK, James MJ, Cleland LG (2005). Diet and rheumatoid arthritis: a review of the literature. Semin Arthritis Rheum..

[45] Gundala NKV, Das UN (2019). Arachidonic acid-rich ARASCO oil has anti-inflammatory and antidiabetic actions against streptozotocin + high fat diet induced diabetes mellitus in Wistar rats. Nutrition..

[46] Gundala NKV, Naidu VGM, Das UN (2017). Arachidonic acid and lipoxinA4 attenuate streptozotocin-induced cytotoxicity to RIN5 F cells in vitro and type 1 and type 2 diabetes mellitus in vivo. Nutrition..

[47] Gundala NKV, Naidu VGM, Das UN (2017). Arachidonic acid and lipoxin A4 attenuate alloxan-induced cytotoxicity to RIN5F cells in vitro and type 1 diabetes mellitus in vivo. Biofactors..

